# Editorial: Prognostic and predictive factors in autoimmune connective tissue disorders, volume II

**DOI:** 10.3389/fimmu.2026.1837711

**Published:** 2026-04-10

**Authors:** Ilaria Mormile, Francesca Wanda Rossi, Mohammed Osman

**Affiliations:** 1 Department of Translational Medical Sciences, University of Naples Federico II, Naples, Italy; 2 Center for Basic and Clinical Immunology Research (CISI), University of Naples Federico II, Naples, Italy; 3 World Allergy Organization (WAO) Center of Excellence, Naples, Italy; 4 Division of Rheumatology, Department of Medicine, University of Alberta, Edmonton, AB, Canada

**Keywords:** autoimmune diseases, autoimmunity, connective tissue disorders, inflammatory arthritis, rheumatoid arthritis, systemic lupus erythematosus, systemic sclerosis

Autoimmune connective tissue diseases comprise a heterogeneous group of complex rheumatologic conditions characterized by potential involvement of multiple organs and systems, often requiring a multidisciplinary management approach. Owing to their broad phenotypic variability, the clinical course of these diseases remains highly unpredictable, ranging from indolent presentations to rapidly progressive and severe forms. Despite substantial advances in diagnostic strategies and therapeutic options over the past decades, the limited availability of reliable biomarkers and prognostic indicators still hampers the ability to accurately predict disease progression and to prevent the development of severe, potentially life-threatening manifestations.

In this Research Topic, we sought to gather the most recent evidence on emerging research directions aimed at predicting clinical phenotypes, long-term outcomes, and guiding the implementation of tailored therapeutic strategies. The contributions included recent advances in immunology and rheumatology, with particular emphasis on their relationship with emerging risk factors and more refined patient stratification. By integrating these perspectives, this Research Topic aimed to enhance the precision of predictive models for treatment response and prevention strategies in autoimmune connective tissue diseases, while also clarifying the potential benefits of optimizing both risk factor management and therapeutic decision-making.

During the submission period of this Research Topic, 17 manuscripts were received; of these, 10 were rejected and 7 were ultimately accepted for publication. The Research Topichas been viewed approximately 22,000 times, reflecting the strong interest of the scientific community in this field and its clear relevance for patient care.

Some authors focused on systemic lupus erythematosus (SLE), a complex autoimmune disease with protean clinical manifestations and variable severity. Anti-dsDNA antibodies are considered a key biomarker in this disease with both diagnostic and prognostic value. Lu et al. performed a comparative evaluation of three anti-dsDNA antibody detection methods (i.e., indirect immunofluorescence (IIF), digital liquid chip method (DLCM), chemiluminescence immunoassay (CLIA), and their combinations) to investigate their sensitivity, specificity, accuracy, and positive and negative predictive values in SLE. The authors confirmed that each method performed well individually, even though enhanced diagnostic sensitivity was observed when combining the different assays to detect anti-dsDNA. In addition, the authors found that DLCM showed better correlation with composite disease activity scores (e.g. SLEDAI-2K), while IIF and CLIA had potential for renal-specific prognostication.

Zheng et al. focused on immune thrombocytopenia (ITP), the most common hematological presentation of SLE, which can be associated with a severe clinical scenario with a higher incidence of major bleeding compared to the primary forms. The authors evaluated the predictive value of SLE related ITP therapeutic outcomes using the systemic immune-inflammation index (SII; platelet count × neutrophil/lymphocyte count at diagnosis), neutrophil-to-lymphocyte ratio (NLR), and complement 3. The findings showed that NLR was positively associated with favorable treatment outcomes, whereas SII and complement C3 were negatively associated, suggesting that these two readily available clinical biomarkers maybe utilized for assessing therapeutic efficacy in patients with SLE.

Other authors in this Research Topic focused on patients with severe organ involvement related to inflammatory arthritis. Ma et al. evaluate a large cohort of 976 patients with rheumatoid arthritis (RA) to identify potential risk factors for the development of combined interstitial lung disease (ILD) and emphysema (CPFE). In their cohort, almost half of the patients developed ILD (42.4%), with 7.6% presented with CPFE. Risk factors for CPFE were identified in male gender, cigarette smoking, occupational exposure to dust, high ILD score (e.g. HRCT >20% of lung parenchyma), high rheumatoid factor titers, and the presence of anti-SSA antibodies. In addition, patients with CPFE showed higher levels of IgG and IgM autoantibodies targeting primary human bronchial epithelial cells – suggesting that *in situ* pulmonary inflammation may contribute to its pathogenesis.

In an extensive review article, Wang et al. assessed the utility of severe extra-articular complication of RA to prognostically assess the risks associated with rheumatoid vasculitis (RV) – an uncommon – yet lethal complication of RA. The authors identified concurrent infections and elevated anti-cyclic citrullinated peptide (anti-CCP) antibody titers as potential adverse prognostic factors. The latter result in particular aligns with existing studies suggesting that the deposition of immune complexes, formed by the binding of autoantibodies (rheumatoid factor and anti-CCP antibodies) to their target antigens on the vascular wall, significantly contributes to the pathogenesis of this condition. The authors also revised predominant clinical manifestations reported in the literature, revealing that RV typically presents with isolated organ involvement mainly affecting the skin and central nervous system. Finally, the authors provide support for the use of biologic disease-modifying antirheumatic drugs in the management of RV.

Other forms of vasculitis were also closely investigated in this Research Topic. Namely, Kudraszew et al. conducted an extensive review of the literature to synthesize current evidence related to the role of calprotectin as a biomarker and the pathogenesis in giant cell arteritis – particularly as it is relatively independent from the IL-6 pathway. They concluded that serum calprotectin be a promising biomarker with potential utility in the diagnosing disease, assessing ongoing activity, and potentially guiding treatment decisions - especially in patients receiving tocilizumab.

Shifting the focus to vaccination research in inflammatory arthritis, another study by Wroński et al. evaluated gene expression and concentration levels of 13 proteins involved in cellular immunity (i.e, IL-2, TNF, Fas, FasL, TRAIL, LT-a, cathepsins, perforin, and granzymes) following the COVID-19 booster vaccination in patients with inflammatory arthritis treated with different immunomodulatory drugs. These results suggested that COVID-19 vaccination elicits a reduced cellular response in patients compared with controls, particularly in those treated with glucocorticoids, methotrexate, and biologic/targeted synthetic disease-modifying antirheumatic drugs, except IL-17 inhibitors, which did not affect the cellular response.

Finally, an elegant study by van Eeden et al., systematically assessed severe fatigue, a frequently overlooked condition in autoimmune rheumatic diseases, and its association with clinical and laboratory features in systemic sclerosis. In particular, the authors reported that severe fatigue resembling myalgic encephalomyelitis was associated with decreased nailfold capillary density, diminished lung function, and elevated galectin-9 levels with increased markers linked with vascular injury (e.g. VEGF-A) – suggesting that severe and chronic fatigue may represent more than patient symptoms, and may reflect a different disease state related to the pathogenesis of SSc.

The recent advances reported in the Research Topic promote adapting personalized medicine-based approaches, in which disease-related therapeutic decisions may be customized using specific hitherto biomarkers. Future studies are required to translate some of these findings into clinical practice which ultimately may result in improved patient outcomes.

